# Parallel detection of multiple zoonotic parasites using a real-time fluorogenic loop-mediated isothermal amplification-based quadruple-sample microfluidic chip

**DOI:** 10.3389/fmicb.2023.1238376

**Published:** 2023-09-26

**Authors:** Yu-Xin Chen, Yi-Rong Lou, Li-Jun Duan, Qian-Jin Zhou, Zhong-Jie Xu, Fang-Jie Chen, Hong-Xian Chen, Gui-Zong Xu, Ai-Fang Du, Jiong Chen

**Affiliations:** ^1^State Key Laboratory for Managing Biotic and Chemical Threats to the Quality and Safety of Agro-products, Ningbo University, Ningbo, China; ^2^School of Marine Sciences, Ningbo University, Ningbo, China; ^3^Ningbo Haishu District Animal Husbandry and Veterinary Medicine Technical Management Service Station, Ningbo, China; ^4^Institute of Preventive Veterinary Medicine, Zhejiang Provincial Key Laboratory of Preventive Veterinary Medicine, College of Animal Sciences, Zhejiang University, Hangzhou, China; ^5^Key Laboratory of Aquacultural Biotechnology Ministry of Education, Ningbo University, Ningbo, China

**Keywords:** zoonotic parasite, microfluidic chip, loop-mediated isothermal amplification, low reagent consumption, multiple detection

## Abstract

Zoonotic parasites pose significant health risks globally. In the present study, we combined a microfluidic chip with loop-mediated isothermal amplification (on-chip LAMP) to detect five zoonotic parasites: *Toxoplasma gondii*, *Cryptosporidium parvum*, *Cryptosporidium hominis*, *Clonorchis sinensis*, and *Taenia solium*. This method enabled the simultaneous parallel analysis of five genetic markers from a maximum of four samples per chip. The on-chip LAMP assay was conducted in a highly automated format *via* the addition (by pipetting) of each sample in a single operation. The reaction was performed in volumes as low as 5 μL at a temperature of 65°C for 60 min, achieving limits of detection ranging from 10^−2^ to 10^−3^ pg./μL of recombinant plasmid DNA. All the time-to-positive values were less than 40 min, and almost all the coefficients of variation were less than 10%, even when using limit of detection concentrations for multiple pathogens, indicating robust reproducibility among replicates. The clinical sensitivity and specificity for detecting 135 field samples were 98.08 and 97.59%, respectively, compared with traditional biological methods, indicating good applicability in the detection of field samples. This on-chip LAMP assay allows for low reagent consumption, ease of operation, and multiple analyses of samples and genetic targets, and is applicable for on-site detection and the routine monitoring of multiple zoonotic parasites.

## 1. Introduction

Parasitic zoonoses are caused by parasites that infect vertebrates, including humans (Dawson, 2005). More than 350 agents that are infectious to humans are parasites (Torgerson and Macpherson, 2011), and nearly 90 parasitic zoonoses have been recorded nationwide in China (Chen et al., 2020). *Toxoplasma gondii, Cryptosporidium* spp.*, Taenia* spp., and *Clonorchis sinensis* present high infection risks and have been included in the census of consecutive parasites in China (Qian et al., 2021). These four parasites are highly prevalent globally as well as in China. For example, globally, 33.8% of pregnant women have latent *T. gondii* infections (Rostami et al., 2020); *Cryptosporidium* spp. infection has been documented in all provinces of China (Liu et al., 2020); approximately 150,000 individuals are affected by *C. sinensis* in East Asia (Qian et al., 2012); and the global infection rate of cysticerci (the cercariae form of *T. solium*) is 3%, but it has escalated to 17% in Central America, South Africa, and East Asia (Coral-Almeida et al., 2015). The popularity of companion animals and the widespread trade and consumption of raw foods, such as freshwater products and meats, have resulted in a persistent and high incidence of infections caused by *T. gondii* and *Cryptosporidium* spp. (Dorny et al., 2009; Berrouch et al., 2020). Additionally, diseases caused by *C. sinensis* and *T. solium* are also on the rise (Dorny et al., 2009; Li et al., 2014; Qian et al., 2021). Furthermore, the concentration of foods for sale in farmers’ markets has raised the risk of cross-contamination of various foodborne parasites between different food items, both abroad and in China (Dorny et al., 2009; Carrique-Mas and Bryant, 2013; Ng-Nguyen et al., 2018; Openshaw et al., 2018; Li et al., 2020; da Costa Dantas et al., 2023). Therefore, the effective elimination of infectious agents from sources such as food, water, and companion animals is crucial for controlling parasitic zoonoses. Additionally, the development of a universal method for the rapid and efficient detection of various parasites would greatly benefit parasite control.

Current methods for the detection of parasitic pathogens mainly depend on etiology, immunology, or molecular biology. Etiological methods based on morphological observations present inherent complexities that can often only be anticipated with professional skills and experience (Ryan et al., 2017). Additionally, this method is often associated with a low detection rate, a high missed detection rate, and a certain false-positive rate (Ahmed and Karanis, 2018). Immunological methods, particularly enzyme-linked immunosorbent assays and indirect fluorescent antibodies, are commonly employed in the surveillance of certain parasitic diseases (Dubey et al., 2020). However, these methods tend to exhibit limited sensitivity and require a relatively high sample volume, often failing to meet the clinical threshold for many protein biomarkers (Satija et al., 2016; Hosseini et al., 2018). Polymerase chain reaction (PCR)-based nucleic acid testing is highly efficient for the laboratory diagnosis of parasitic diseases (Zhang et al., 2020; García-Bernalt Diego et al., 2021). However, these methods are laborious and redundant for the detection of multiple pathogens. Additionally, these methods require sophisticated and expensive equipment (García-Bernalt Diego et al., 2021).

Microfluidic chips, also known as lab-on-a-chip, integrate sample pretreatment, biological separation, biochemical reactions, and signal analysis into chips of a few square centimeters. Their micrometer-scale structures allow precise control of fluid flow in microchannels, enabling the miniaturization and automation of comprehensive analyses (Pattanayak et al., 2021). Isothermal amplification methods have recently accelerated the development of microfluidic chips (Xiao et al., 2023). The isothermal amplification-based microfluidic chip method offers a combination of the advantages of isothermal amplification for detecting nucleic acid, such as high specificity and sensitivity, and short detection time, as well as the benefits of microfluidic chips, including controllable liquid flow, efficient use of samples and reagents, increased analysis speed, and low cost (Pattanayak et al., 2021; Pérez-Grisales et al., 2021; Atceken et al., 2022). Several microfluidic chips have been used to detect various veterinary pathogens, including viruses, bacteria, and parasites (Mao et al., 2018; Malpartida-Cardenas et al., 2019; Zhou et al., 2020; Ye et al., 2021). To date, only a few microfluidic systems have been utilized on-site, and there is an urgent need for flexible, versatile, multitarget, and multisampling chips (García-Bernalt Diego et al., 2021). Thus, methods of examining multiple samples and indicators in microfluidic systems would make these systems more practical.

The objective of this study was to establish a multiple-sample and multiple-target detection system for *T. gondii*, *C. parvum*, *C. hominis*, *C. sinensis*, and *T. solium*, comprising four samples and five indicators. The performance of this system was verified using clinical samples and compared with conventional microbiological methods and PCR assays.

## 2. Materials and methods

### 2.1. Zoonotic parasitic and bacterial isolates and culture conditions

Nineteen pathogenic microorganisms were used in this study, including 15 parasitic isolates and four bacterial strains (Supplementary Table S1). *T. gondii* type II strain ME49 and *Cryptosporidium* spp. (including *C. parvum*, *C. hominis*, *C. suis*, and *C. baileyi*) were kindly provided by Prof. Du Aifang (Zhejiang University), and maintained as described in Supplementary Table S1. No isolates of *C. sinensis* and *T. solium* were kept in our laboratory; instead, their gDNA were kindly provided by Prof. Du Aifang and verified by PCR methods. Information and culture conditions for other non-targeted microorganisms can be found in Supplementary Table S1.

### 2.2. Primer determination

In this study, five species of zoonotic parasites, *T. gondii*, *C. parvum*, *C. hominis*, *T. solium*, and *C. sinensis* were selected as the target microorganisms. All the specific loop-mediated isothermal amplification (LAMP) primer sets used in this study were obtained from the published literature. The primer sequences are listed in Table 1. Primer performance, that is methodological specificity, methodological sensitivity, and real-time amplification plots, were verified using optimized LAMP conditions as described previously (Zhou et al., 2014).

**Table 1:** LAMP primers used in this study.

Species	Target sequence	Primer set	Primers	Sequence (5′-3′)	Source
*Toxoplasma gondii*	repeat region (GenBank No. AF146527)	Tgon-repeatregion	Tgon-repeatregion-F3	CCACAGAAGGGACAGAAGTC	Zhang et al. (2009)
			Tgon-repeatregion-B3	TCCGGTGTCTCTTTTTCCAC	
			Tgon-repeatregion-FIP	TCCTCACCCTCGCCTTCATCTAGGACTACAGACGCGATGC	
			Tgon-repeatregion-BIP	TGGTTGGGAAGCGACGAGAGTTCCAGGAAAAGCAGCCAAG	
			Tgon-repeatregion-LF	TCCAAGACGGCTGGAGGAG	
			Tgon-repeatregion-LB	CGGAGAGGGAGAAGATGTTTCC	
*Cryptosporidium parvum*	gp60 (GenBank No. AB237136)	Cpar-gp60	Cpar-gp60-F3	TCGCACCAGCAAATAAGGC	Bakheit et al. (2008)
			Cpar-gp60B3	GCCGCATTCTTCTTTTGGAG	
			Cpar-gp60-FIP	ACCCTGGCTACCAGAAGCTTCAGAACTGGAGAAGACGCAGAA	
			Cpar-gp60-BIP	GGCCAAACTAGTGCTGCTTCCCGTTTCGGTAGTTGCGCCTT	
			Cpar-gp60-LF	GTACCACTAGAATCTTGACTGCC	
			Cpar-gp60-LB	AACCCACTACTCCAGCTCAAAGT	
*Cryptosporidium parvum* *Cryptosporidium hominis*	SAM-1 (GenBank No. AY161084, XM_662396)	Cpar-SAM1	Cpar-SAM1-F3	ATTTGATRGACAAAGAAACTAG	Bakheit et al. (2008)
			Cpar-SAM1-B3	CGATTGACTTTGCAACAAG	
			Cpar-SAM1-FIP	TTGCGCCCTGTTAATCCAGCATTAATTAATCCATCTGGCAGRTTT	
			Cpar-SAM1-BIP	TTGTAGATACATACGGAGGATGGGTCTACTTTAGTTGCATCTTTCC	
			Cpar-SAM1-LF	CTGCTGGCCCMCCAATTG	
			Cpar-SAM1-LB	CATGGRGGTGGTGCATTTAG	
*Clonorchis sinensis*	cathepsin B3 (GenBank No. AY273803)	Cpar-SAM1	Csin-cathepsin-F3	CGGCTACAAATCTGGTGTGT	Cai et al. (2010)
			Csin-cathepsin-B3	GCGGTGACCTCATCTTCAA	
			Csin-cathepsin-FIP	TCTTCCCCCCAGCCCAAAATG-TTTCCATTCTGATGGCACGC	
			Csin-cathepsin-BIP	ATTCATGGAACGATGGCTGGGG-CTCATTTTTTCCGCGCAACA	
			Csin-cathepsin-Lp	CGAATGGCATGACCACCAAGAA	
*Taenia solium*	cathepsin L-like cysteine protease (clp) (GenBank No. AB441815)	Tsol-clp	Tsol-clp-F3	GAGGTCAAAAATCAGGTGAGAT	Nkouawa et al. (2009)
			Tsol-clp-B3	AATGCTCCTGACTTGGTT	
			Tsol-clp-FIP	AGGTGCTTTCACAATAGTCCCCTGCGTCATAGGTCTTGC	
			Tsol-clp-BIP	TAGTCGTTGCTTCGATAGAGCTCGCTGATATCTAGGCTAATGCTG	

### 2.3. Sample collection

A total of 135 samples were collected from Ningbo City, Zhejiang Province, China, including 50 pig feces, 40 cow feces, 20 pieces of pork, and 25 freshwater fish. The fecal matter consisted of rectal collections or fresh discharge from animals, whereas the pork and fresh fish samples were harvested from local farms or slaughterhouses. The samples were divided into three equal parts. One aliquot was used for parasite identification using conventional microbiological methods. Another aliquot was used for gDNA extraction in the microfluidic chip analysis and PCR assays.

### 2.4. DNA preparation

To extract gDNA from *T. gondii*, *Cryptosporidium* spp., and *G. plecoglossi*, 100 μL of each protozoal suspension was subjected to three rounds of freeze-thawing for 5 min in liquid nitrogen, followed by incubation in a 65°C water bath for 5 min. The treated samples were then centrifuged at 12,000 × g for 5 min, as described by Wang et al. (2014). Genomic DNA was extracted using a Genomic DNA Extraction Kit (TaKaRa, Dalian, China). The gDNA of *A. simplex* was extracted from third-stage larvae using the EZNA Tissue DNA Kit (Omega Bio-Tek, Norcross, GA, United States). The gDNA of the shrimp was extracted from field sample tissues using a Universal Genomic DNA Extraction Kit (TaKaRa). For bacterial isolates, gDNA was extracted from liquid cultures of single clones using a Bacterial DNA Kit (Omega Bio-Tek). All DNA was quantified using a NanoDrop® Spectrophotometer 2000 (Thermo Fisher Scientific, Waltham, MA, United States) and stored at −80°C for further use.

To construct the recombinant plasmids, specific gene segments of the five zoonotic parasites, including the repeat region for *T. gondii*, *gp60* for *C. parvum*, *SAM1* for *C. parvum* and *C. hominis*, cathepsin L-like cysteine protease gene (*clp*) for *T. solium*, and cathepsin B3 gene for *C. sinensis* were cloned into the pMD19-T simple plasmid (TaKaRa) by PCR using primers Tgon-repeatregion-F3/Tgon-repeatregion-B3, Cpar-gp60-F3/Cpar-gp60-B3, Cpar-SAM1-F3/Cpar-SAM1-B3, Tsol-clp-F3/Tsol-clp-B3, and Csin-cathepsin-F3/Csin-cathepsin-B3. After sequencing, the recombinant plasmids (named pMD-Tgon-repeatregion, etc.) were extracted using the Plasmid Mini Kit I (Omega Bio-Tek). Serial dilutions of recombinant plasmid DNA ranging from 10^2^ to 10^−4^ pg./μL were used to measure the limits of detection (LODs) of the on-chip LAMP assay.

### 2.5. Chip design and fabrication, and experimental equipment

**Figure 1:**
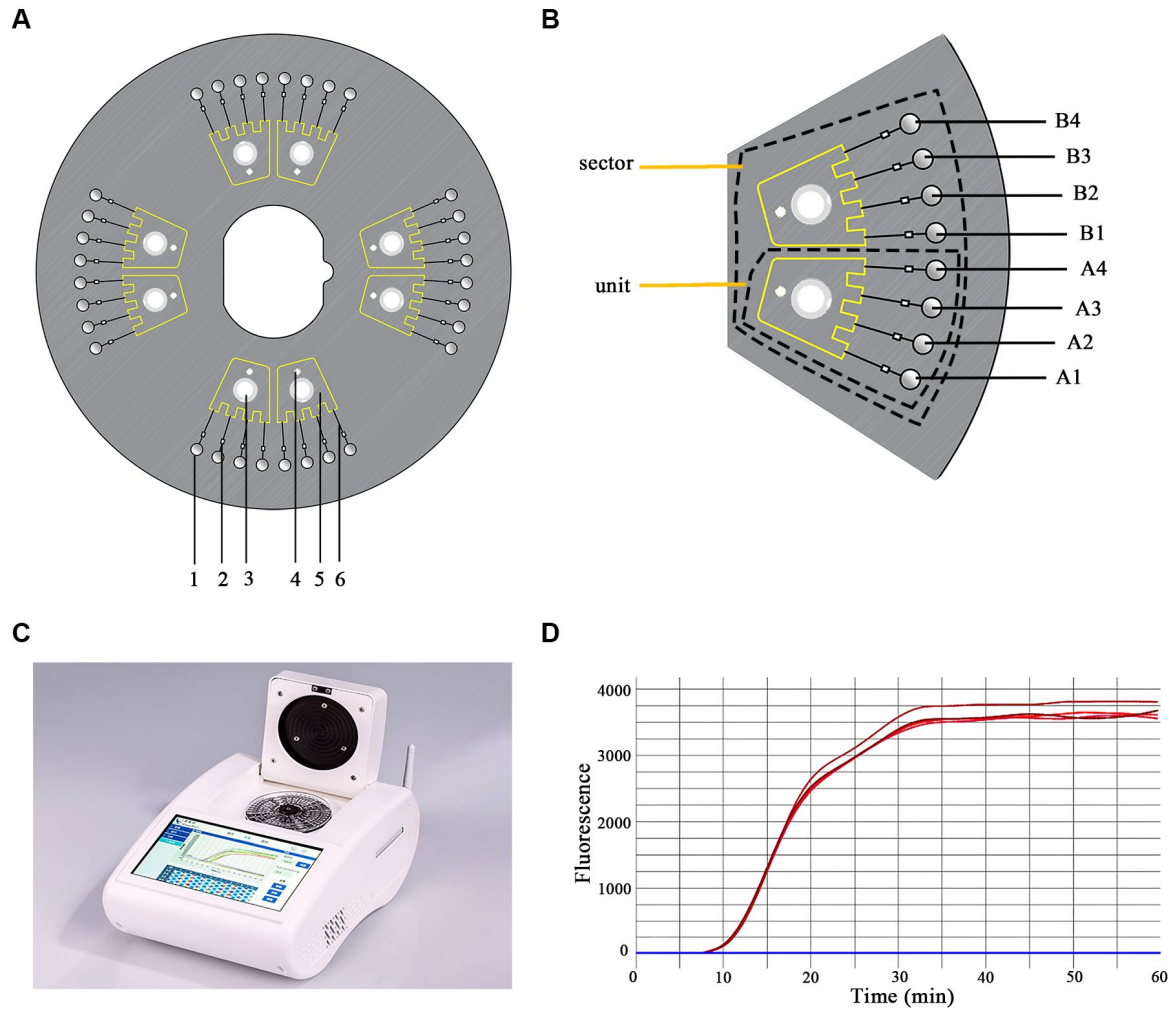
Schematic representation of the chip structure. **(A)** The structure of the microfluidic chip. 1. reaction well; 2. ball valve; 3. inlet hole; 4. vent hole; 5. liquid storage chamber; 6. capillary channel. **(B)** One sector containing two uniform units. A1–A4 and B1–B4 are the reaction wells. **(C)** The equipment used in this study (iGeneTec™ SC-MA2000). **(D)** The fluorescence curve of the LAMP reaction.

In this study, a dished microfluidic chip was divided into four sectors, each containing two separate and uniform units with the same structure (Figures 1A,B). Each unit consisted of four reaction wells connected to a liquid storage chamber *via* capillary channels and ball valves. Ball valves were located on the side near the reaction wells of the capillary channels to avoid cross-contamination. Additionally, inlet and vent holes were set up (Figure 1B). Primer sets were spotted onto corresponding reaction wells at specific ratios. The chip was then sealed with sealing film. The premixed primer-free reaction system and template gDNA were mixed to a total volume of 25 μL, which was added to the inlet holes and dispatched into the reaction wells *via* centrifugal force (Figure 1A). The reaction was conducted at 65°C for 60 min.

Each sector of the chip was designed to analyze five parasites (*T. gondii*, *C. parvum*, *C. hominis*, *C. sinensis*, and *T. solium*) in one sample. Thus, the chip could detect four samples simultaneously. Using one sector as an example, the primer layout was as follows: primers for *T. gondii*, *C. parvum*, *C. hominis*/*C. parvum*, *C. sinensis*, and *T. solium* were spotted into reaction wells A1, A2, A3, B1, and B2; B3 was used as a negative control. The primer layouts for the other sectors were the same. Each set of primers were composed of 0.2 pmol each of the primers F3 and B3, 1.6 pmol each of the primers FIP and BIP, and 0.4 pmol each of the primers LF and LB. The primers used are listed in Table 1.

The main device matching the Chip was equipped with a temperature control module, a centrifugal module, and a real-time fluorescent acquisition and analysis unit (Figure 1D), which enabled a user-friendly “sample-in result-out” operation. The equipment (Product Model: iGeneTec™ SC-MA2000) was provided by iGeneTec (Ningbo, China) and had dimensions of 280 × 200 × 135 mm (length × width × height) (Zhou et al., 2020) (Figure 1C).

### 2.6. Real-time on-chip LAMP assay

The reaction reagents in the reaction system of the on-chip LAMP assay included 10 mM KCl, 6.5 mM MgSO_4_, 10 mM (NH_4_)_2_SO_4_, 0.1% Triton X-100, 1.6 M betaine (Sigma Aldrich, St. Louis, MO, United States), 20 mM Tris–HCl (pH 8.8), 1.4 mM dNTPs, 1 × EvaGreen dye (Biotium Inc., CA, United States), and 8 U Bst DNA polymerase large fragment (New England Biolabs, Beverly, MA, United States).

A 25-μL reaction mixture containing 14.8 μL reaction reagents, 2 μL DNA template at the desired concentration, and 8.2 μL DNA-free ddH_2_O was loaded into each unit of the sector *via* the inlet hole using a pipettor. Both the inlet and vent holes were immediately sealed with film. The chip was anchored to the equipment (iGene™ SC-MA2000; iGeneTec, Ningbo, China). The reaction conditions were set at 65°C for 60 min, and the working procedure was set as low-speed centrifugation for 5 s at 1600 rpm and high-speed centrifugation for 10 s at 4600 rpm. Fluorescence signals were captured every 30 s using the endpoint detection method (Zhou et al., 2014).

### 2.7. Specificity of the on-chip LAMP assay

In this study, the specificity of the on-chip LAMP assay was determined by testing 19 pathogenic microorganisms, including the five parasitic targets. The template for each microorganism was set at a gDNA concentration of 10^1^ pg./μL. During the experiment, one primer set was pre-immobilized on seven units of a chip, with the remaining unit serving as a blank control that contained no primers. A DNA template was added to each primer-immobilized unit and ddH_2_O was added to one of the units as a negative control. Thus, five different kinds of chips, each pre-immobilized with a unique set of primers, were used for the on-chip LAMP assay specificity tests. All experiments were performed in triplicate.

### 2.8. The limits of detection of the on-chip LAMP assay

The LODs of the on-chip LAMP assay were verified using recombinant plasmids ranging from 10^2^ to 10^−4^ pg./μL which had been prepared in advance. The primer layout on the chip was similar to that described in Section 2.7. The only difference was that all eight units on the chip were pre-immobilized with a unique primer set. Sterile DNA-free ddH_2_O was used as the negative control. The time-to-positive value (Tp) was used to denote the initiation of efficient amplification in the on-chip LAMP assay. The coefficient of variation (CV), which is calculated as the ratio of the standard deviation (SD) to the mean (CV = SD/average), was used to evaluate the relative dispersion of Tp. A CV value of less than 10% indicates good reproducibility.

### 2.9. Reproducibility of LAMP reactions across chips

To further evaluate the reproducibility of the LAMP reactions across chips, assays were conducted using 1 pg./μL DNA from each recombinant plasmid as the template. A unique primer set was pre-immobilized in the reaction wells of half the chip. During the experiment, eight reaction wells from one sector were pipetted with the same amount of DNA from one recombinant plasmid, while the wells from the other sector were added using ddH_2_O as a negative control. All experiments were performed in triplicate.

### 2.10. Parallel detection of multiple parasitic DNA

To assess the performance of on-chip LAMP in detecting multiple parasitic pathogens in parallel, two DNA combinations were formed using the six recombinant plasmids mentioned above at the desired concentrations (10-fold higher than the LOD concentration of the on-chip LAMP assay), that is, DNA Combination 1 (containing the pMD-Tgon-repeatregion, pMD-Chom-SAM1, and pMD-Tsol-clp) and DNA Combination 2 (containing the pMD-Tgon-repeatregion, pMD-Cpar-gp60, pMD-Cpar-SAM1, pMD-Tsol-clp, and pMD-Csin-cathepsin). The primer set layout on the chip followed the description in Section 2.5, which enabled simultaneous detection of the five target parasitic pathogens in each sector of the chip. All experiments were performed in triplicate.

### 2.11. Conventional biological methods

In traditional biological diagnostics, various parasites are diagnosed using distinct methods. In China, *T. gondii* is primarily clinically diagnosed using the indirect fluorescent antibodies test, the steps of which are outlined in the Chinese standard GB/T 18448.2–2001. *Cryptosporidium* spp. are diagnosed through fecal examination using the modified acid-fast staining method in accordance with the Chinese health industry standard WS/T 487–2016. Cysticercariae (*T. solium*) are primarily diagnosed through tongue palpation combined with enzyme-linked immunosorbent assay in pig slaughterhouses, following the Chinese standard GB/T 18644–2002. *C. sinensis* in diseased fish is detected by microscopic examination of fish flesh following Na et al. (2020).

### 2.12. PCR assays

*T. gondii* can be detected at the SAG2 locus using nested PCR assays employing two pairs of primers (SAG2.F4: 5′-GCTACCTCGAACAGGAACAC-3′ and SAG2.R4: 5′-GCATCAACAGTCTTCGTTGC-3′; SAG2.F: 5′-GAAATGTTTCAGGTTGCTGC-3′ and SAG2.R2: 5′-GCAAGAGCGAACTTGAACAC-3′) (Howe et al., 1997). *Cryptosporidium* spp., *C. sinensis*, and *T. solium* can be detected using conventional PCR assays (Johnson et al., 1995; González Luis et al., 2000; Traub et al., 2009).

### 2.13. Clinical sensitivity and specificity

Conventional biological methods were used as the standard to evaluate the clinical sensitivity and specificity of the on-chip LAMP assays. Assuming a clinical sensitivity and specificity of 100% for conventional biological methods, the clinical sensitivity and specificity of the on-chip LAMP and PCR assays were calculated using the following formulas: clinical sensitivity (%) = true positives/(true positives + false negatives) × 100 and clinical specificity (%) = true negatives/(true negatives + false positives) × 100. Additionally, the 95% confidence intervals (95% CI) were calculated for both clinical sensitivity and specificity.

## 3. Results

### 3.1. Evaluation of the specificity of the on-chip LAMP assay

**Figure 2:**
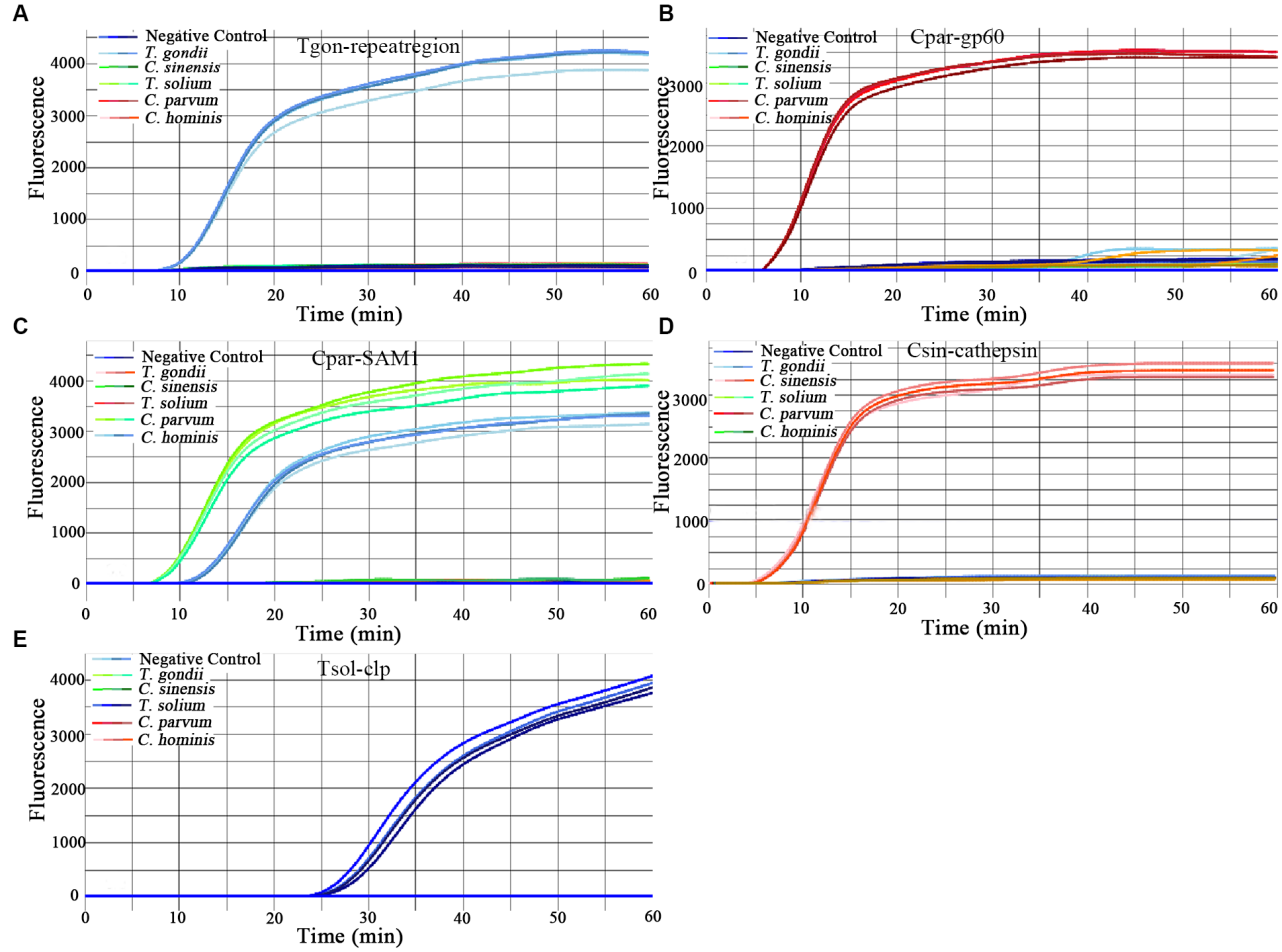
Amplification curves of the on-chip LAMP assays using five primer sets. One primer set was pre-immobilized in seven units of a chip, and the left unit contained no primers. A DNA template with a concentration of 10 pg./μL was added to each primer-immobilized unit, and ddH_2_O was added to one unit as the negative control. The primer sets were as follows: **(A)** Tgon-repeatregion; **(B)** Cpargp60; **(C)** Cpar-SAM1; **(D)** Csin-cathepsin; and **(E)** Tsol-clp.

The evaluation of the specificity of the on-chip LAMP assay included testing for cross-reactivity among the five parasitic targets and reactivity against the gDNA of 14 other pathogenic microorganisms. The results indicated that all primer sets produced positive amplification of their respective target gDNA, displaying a typical sigmoidal amplification curve (Figure 2). Notably, the primer set Cpar-SAM1 produced positive amplification curves when using gDNA from both *C. parvum* and *C. hominis*, whereas the primer set Cpar-gp60 generated a positive amplification curve for *C. parvum* gDNA but not for *C. hominis*. These findings align with those of a previous report (Bakheit et al., 2008) and can aid in distinguishing *C. hominis* and *C. parvum*. Additionally, no positive amplification was detected using gDNA from other pathogens, such as *C. suis*, *C. baileyi*, *T. canis*, *T. spiralis*, *Ichthyophthirius* spp., *A. simplex*, *E. hepatopenaei*, *B. bovis*, *S. japonicum*, *G. plecoglossi*, *S. iniae*, *A. hydrophila*, *V. Parahemolyticus*, and *L. monocytogenes*. Overall, the results indicated good specificity of the different primer sets.

### 3.2. Evaluation of the LODs of the on-chip LAMP assays

**Figure 3:**
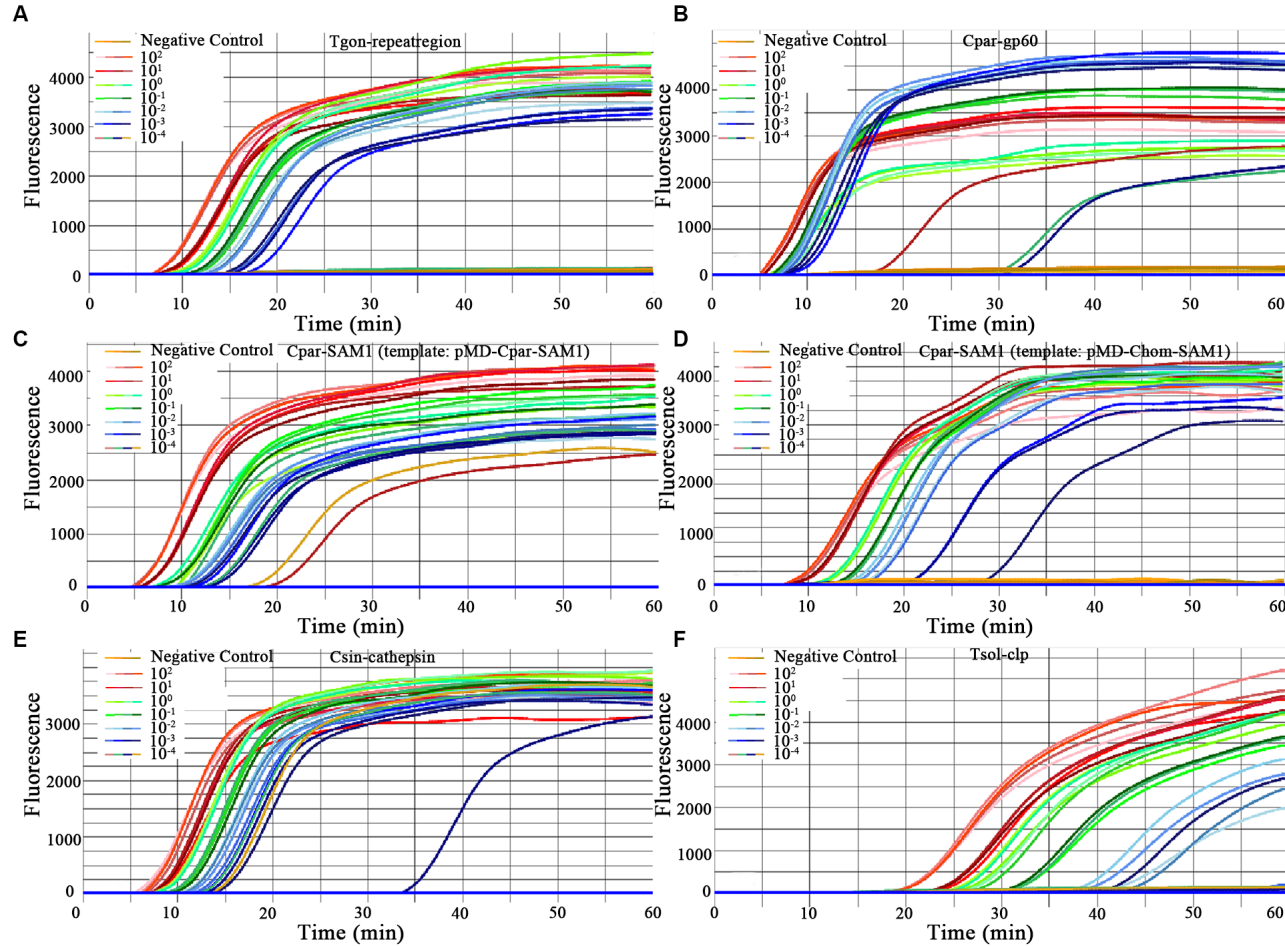
Amplification curves for primer sensitivity test. Each of the eight units on the chip was pre-immobilized with a unique primer set, and different concentrations of the same DNA template were added to each unit (ranging from 10^2^ to 10^−4^ pg./μL DNA for each recombinant plasmid). Sterile, DNA-free ddH_2_O was employed as a negative control. **(A)** Primer set: Tgon-repeatregion, Template: pMD-Tgon-repeatregion; **(B)** Primer set: Cpar-gp60, Template: pMD-Cpar-gp60; **(C)** Primer set: Cpar-SAM1, Template: pMD-Cpar-SAM1; **(D)** Primer set: Cpar-SAM1, Template: pMD-Chom-SAM1; **(E)** Primer set: Csin-cathepsin, Template: pMD-Csin-cathepsin; **(F)** Primer set: Tsol-clp, Template: pMD-Tsol-clp.

**Figure 4:**
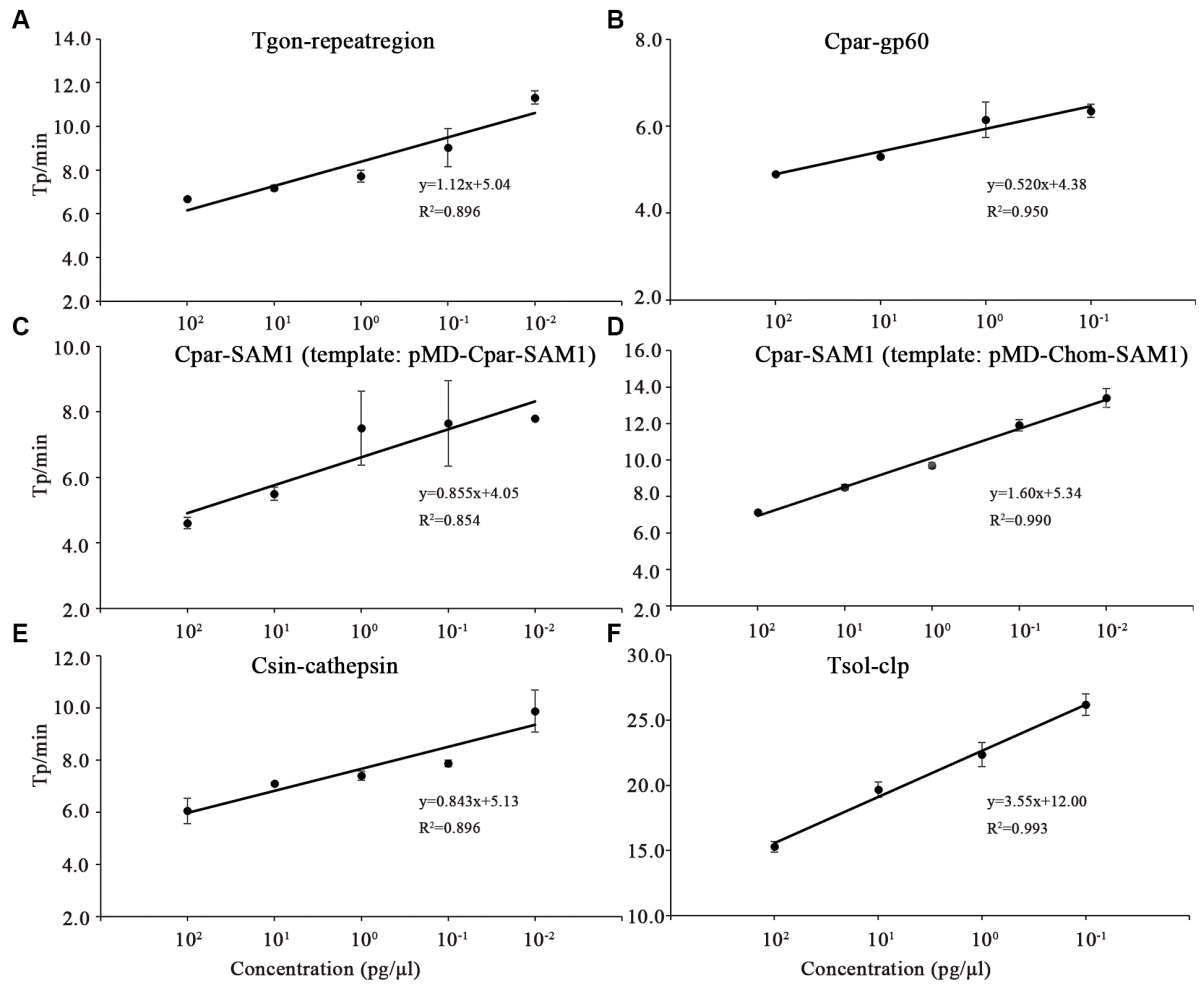
Correlation between the time-to-positive value (Tp) and DNA concentration of the on-chip LAMP reaction. Different concentrations for each recombinant plasmid ranging from 10^2^ to 10^−4^ pg./μL were used in the on-chip LAMP assay. Sterile, DNA-free ddH_2_O was employed as a negative control. **(A)** Primer set: Tgon-repeatregion, Template: pMD-Tgon-repeatregion; **(B)** Primer set: Cpar-gp60, Template: pMD-Cpar-gp60; **(C)** Primer set: Cpar-SAM1, Template: pMD-Cpar-SAM1; **(D)** Primer set: Cpar-SAM1, Template: pMD-Chom-SAM1; **(E)** Primer set: Csin-cathepsin, Template: pMD-Csin-cathepsin; **(F)** Primer set: Tsol-clp, Template: pMD-Tsol-clp.

**Figure 5:**
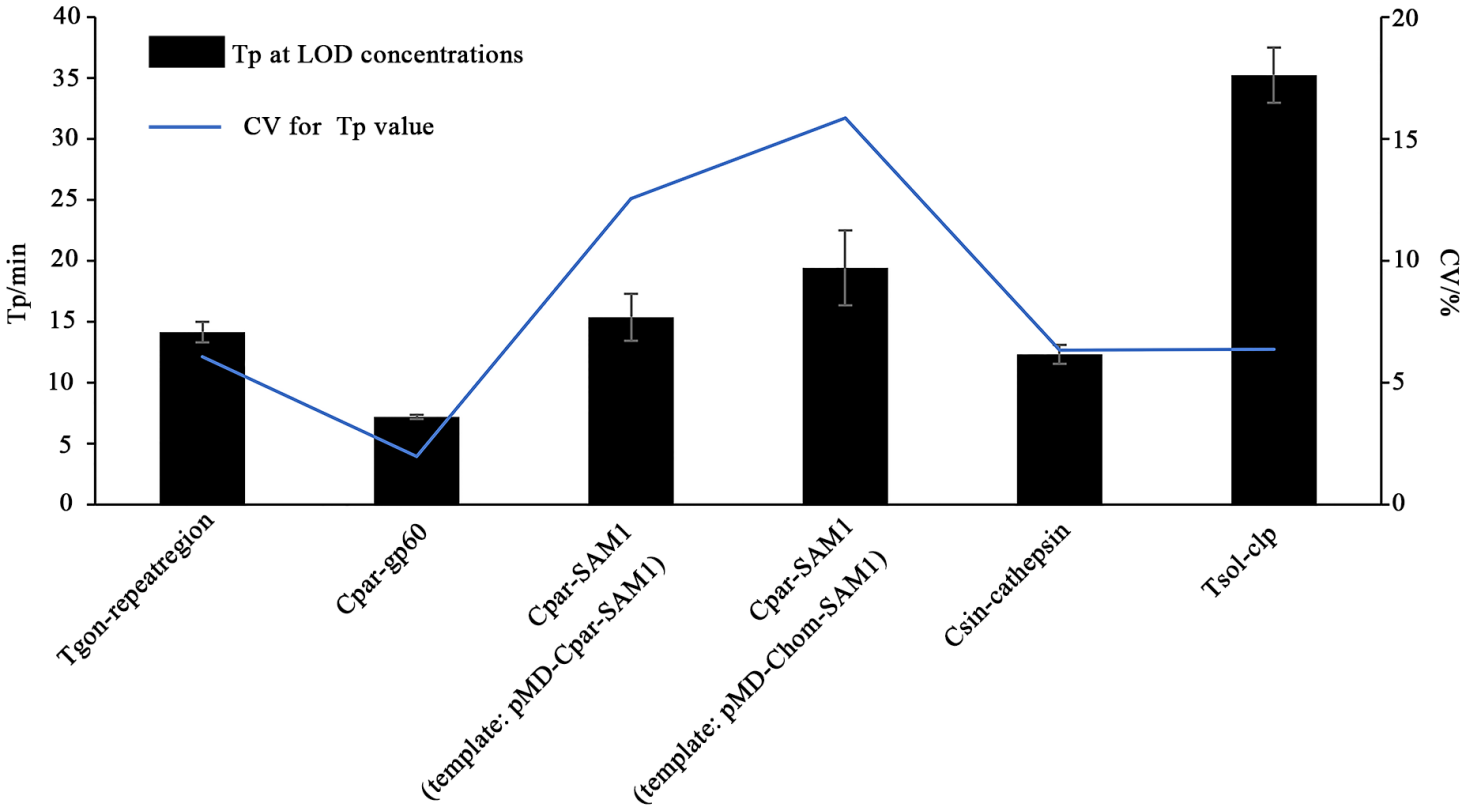
Time-to-positive values (Tp) and their coefficient of variation (CV) obtained from the on-chip LAMP assay using DNA at the LOD concentration as the template. The X-axis represents the primer sets Tgon-repeatregion, Cpar-gp60, Cpar-SAM1, Cpar-SAM1, Csin-cathepsin, and Tsol-clp. The Y-axis on the left indicates the Tp values, while the Y-axis on the right indicates the CV values of the Tp values.

In this study, the LODs of the on-chip LAMP assay were evaluated using recombinant plasmid DNA from five different parasites, diluted 10-fold. The results demonstrated that the LAMP reaction could produce a typical “S” type curve when pMD-Tgon-repeatregion for *T. gondii* was used as the template at various concentrations, including 10^2^, 10^1^, 10^0^, 10^−1^, 10^−2^, and 10^−3^ pg./μL, while no positive amplification was detected when 10^−4^ pg./μL DNA was used as a template (Figure 3A). This revealed that the LOD of the chip for *T. gondii* is 10^−3^ pg./μL recombinant plasmid DNA using primer set Tgon-repeatregion. Furthermore, the LODs of the other four primer sets for the corresponding parasites were as follows: 10^−3^ pg./μL of pMD-Cpar-gp60 for *C. parvum* using primer set Cpar-gp60, 10^−3^ pg./μL of pMD-Cpar-SAM1 and pMD-Chom-SAM1 for *C. parvum* and *C. hominis*, respectively, using primer set Cpar-SAM1, 10^−3^ pg./μL of pMD-Csin-cathepsin for *C. sinensis* using primer set Csin-cathepsin, and 10^−2^ pg./μL of pMD-Tsol-clp for *T. solium* using primer set Tsol-clp (Figure 3). Meanwhile, with a decrease in DNA concentration, the Tp values increased gradually, indicating a typical linear correlation between DNA concentration and Tp value (Figure 4). In addition, all five primer sets showed relatively low Tp values, with almost all Tp values at the LOD concentration being less than 20 min, except for the primer set Tsol-clp, which produced a Tp value of approximately 35 min (Figure 5). The CVs for Tp values were all below 5% when using 10^2^ to 10^−1^ pg./μL DNA from the six recombinant plasmids as the templates (data not shown). However, when the LOD concentration of each recombinant plasmid was used as a template, the CV values fluctuated. For instance, the CV values for the primer sets Cpar-SAM1 and Tsol-clp were close to or exceeded 10% when the LOD concentrations of gDNA from recombinant plasmids pMD-Cpar-SAM1 and pMD-Tsol-clp were used, as shown in Figure 5.

### 3.3. Evaluation of reproducibility of LAMP reactions across chips

**Figure 6:**
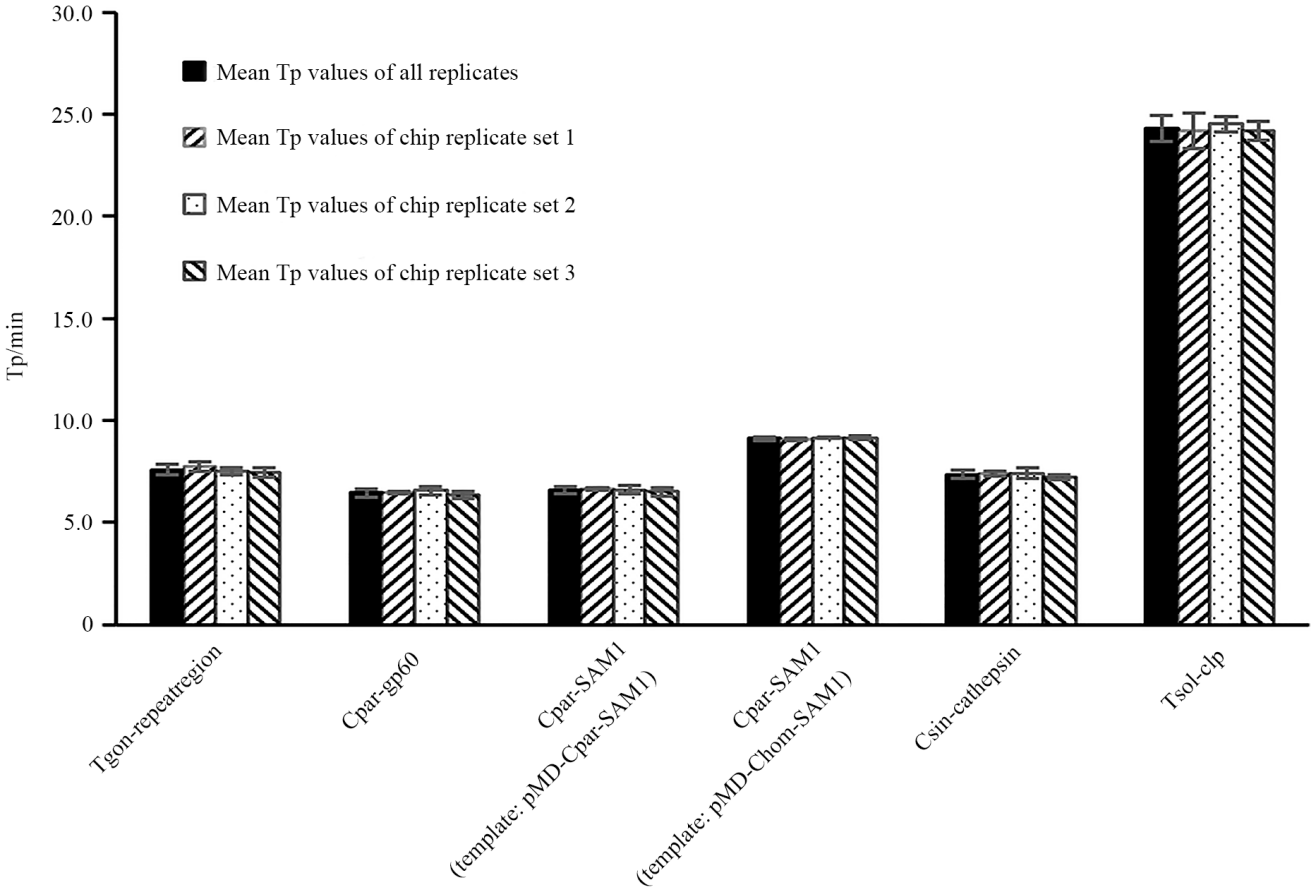
Reproducibility analysis of LAMP reactions within and between chips. The X-axis represents the primer sets Tgon-repeatregion, Cpar-gp60, Cpar-SAM1, Cpar-SAM1, Csin-cathepsin, and Tsol-clp. Eight reaction wells in one sector of the chip were pre-immobilized with a unique primer set and pipetted with a same amount of DNA from one recombinant plasmid. 1 pg./μL DNA from each recombinant plasmid was used as the template.

To evaluate the reproducibility of the on-chip LAMP reactions among the different chips, DNA templates with concentrations close to the median from each recombinant plasmid were used, referring to the dilution gradients mentioned in Section 2.8. At this concentration, all LAMP reactions exhibited stable and smooth positive amplification curves, as shown in Supplementary Figure S1. Most Tp values were less than 10 min, except for the Tsol-clp primer set, which had a mean Tp value close to 25 min (Figure 6). The Tp values for each targeted gene were consistent within and between different chips, and no significant differences were observed between chips (Figure 6; Supplementary Table S2), indicating excellent reproducibility of the on-chip LAMP reactions.

### 3.4. Parallel detection of multiple parasitic DNA

**Figure 7:**
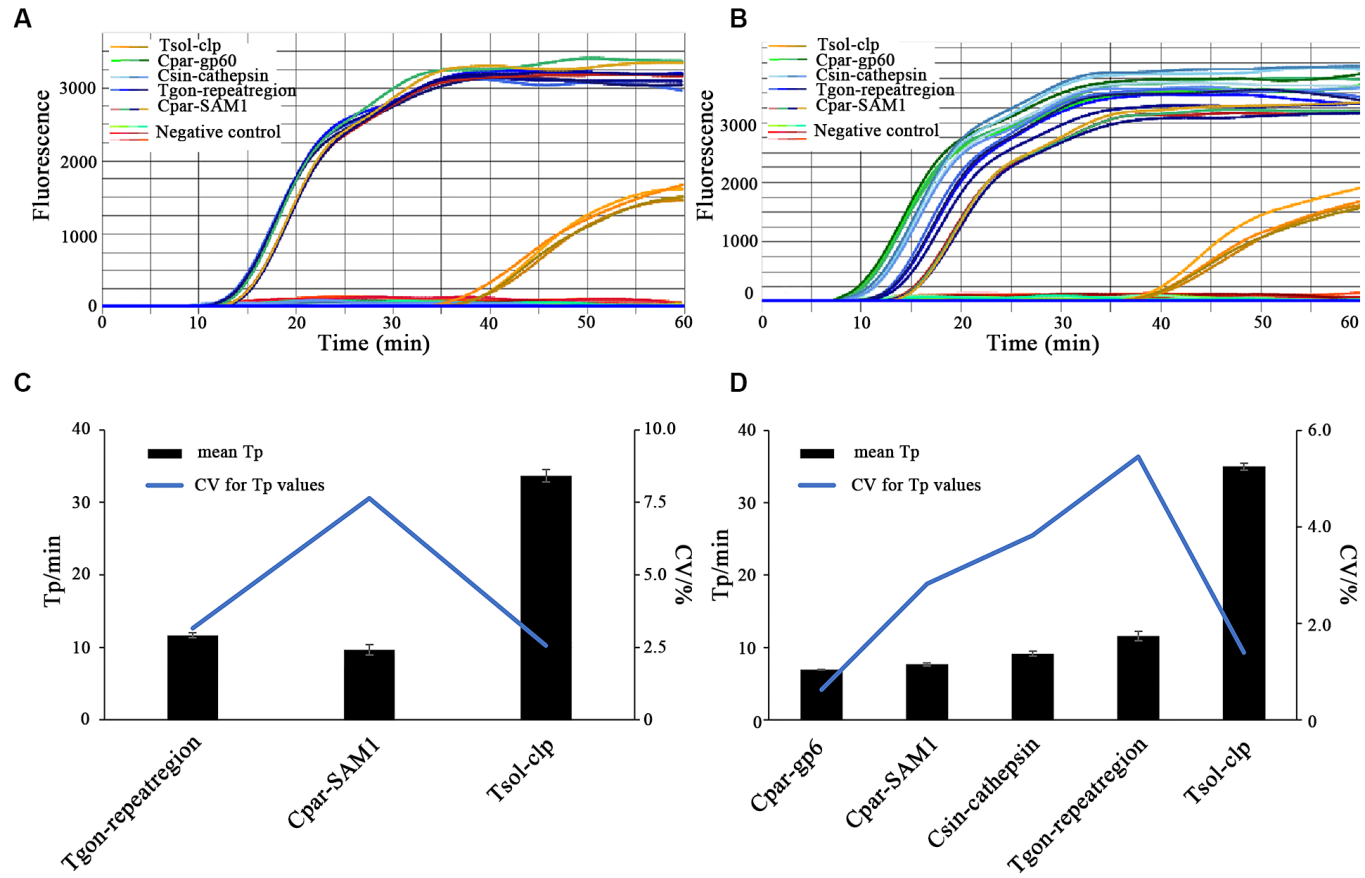
On-chip LAMP testings for parallel detection of multiple parasitic DNA. Primer sets: Tgon-repeatregion, Cpar-gp60, Cpar-SAM1, Csin-cathepsin, and Tsol-clp. **(A,C)** DNA Combination 1 containing recombinant plasmid pMD-Tgon-repeatregion, pMD-Chom-SAM1, and pMD-Tsol-clp; **(B,D)** DNA Combination 2 containing recombinant plasmid pMD-Tgon-repeatregion, pMD-Cpar-gp60, pMD-Cpar-SAM1, pMD-Tsol-clp, and pMD-Csin-cathepsin. 10 × LOD concentration of the recombinant plasmid DNA was used as the template.

All the expected positive amplification curves were observed when DNA Combination 1 and DNA Combination 2 were used as templates (Figures 7A,B). On the chip, four highly consistent positive amplification curves were observed for each target gene (Figures 7A,B). Moreover, most Tp values obtained using each primer set were close to or less than 10 min, except for the primer set Tsol-clp, which had a mean Tp value of approximately 35 min (Figures 7C,D). Despite some fluctuations in the CV values of the individual primer sets when faced with different DNA combinations, all remained below 10% (Figures 7C,D). The above results indicate that each primer set maintained good stability with relatively complex DNA templates.

### 3.5. Clinical sensitivity and specificity of the on-chip LAMP assay

The results showed that *T. gondii*, *Cryptosporidium* spp., and *C. sinensis* were detected in the pig, cattle, and fish samples. Among the 135 samples tested using traditional biological methods, 52 were positive for at least one of the three pathogens, and 83 were negative (Table 2). Among these, 34 samples were positive for *T. gondii*, 17 were positive for *Cryptosporidium* spp., and one was positive for *C. sinensis* (Table 2). On-chip LAMP analysis yielded positive results for 51 of 52 samples that tested positive using conventional biological methods, resulting in a clinical sensitivity of 98.08% (Table 2). Meanwhile, 81 of the 83 samples that tested negative using conventional biological methods also tested negative using on-chip LAMP analysis, resulting in a clinical specificity of 97.59% (Table 2). Additionally, PCR yielded a clinical sensitivity of 94.23% and a clinical specificity of 98.80% (Table 2). These results indicate that the on-chip LAMP method is highly applicable for the diagnosis of field samples.

**Table 2:** Sensitivity and specificity of on-chip LAMP and PCR.

Biological characteristics (*n*)	LAMP on-chip		PCR	
	Positive	Negative	Positive	Negative
Positive(52)	51	1	49	3
Negative(83)	2	81	1	82
Clinical sensitivity	98.08% (89.88–99.66%)		94.23% (84.36%-98.03)	
Clinical specificity	97.59% (91.63–99.34%)		98.80% (93.49–99.79%)	

## 4. Discussion

Currently, significant risk from foodborne zoonotic parasites remains globally (Saijuntha et al., 2021). During the initial phase of infection, parasite levels are typically low, necessitating the use of detection methods with low LODs. Furthermore, mixed infections involving multiple pathogens increase the complexity and difficulty of disease treatment. Therefore, accurate and rapid detection and identification of multiple parasites are crucial to ensure the precise diagnosis of both seasonal and sporadic outbreaks. This study presents an on-chip LAMP method for the detection of five zoonotic parasites, *T. gondii*, *C. parvum*, *C. hominis*, *C. sinensis*, and *T. solium*. The on-chip LAMP system minimizes sample and reagent consumption, enables the simultaneous detection of multiple pathogens, and possesses low LODs (ranging from 10^−2^ to 10^−3^ pg./μL) for recombinant plasmid DNA. Therefore, this method has great potential for the rapid on-site and routine detection of multiple parasites for food safety.

Sensitivity is a critical metric for evaluating the quality of pathogen detection methods. For instance, in the field of conventional LAMP-based detection, Zhang et al. (2009) developed a LAMP method that detects *T. gondii* with an LOD of 1 pg./μL of gDNA, whereas Fallahi et al. (2015) lowered the LOD to 0.1 pg./μL of gDNA. Bakheit et al. (2008) established a LAMP method targeting gp60 to detect *C. parvum* with an LOD of 1 ng/μL of gDNA, and shared a LAMP method targeting SAM-1 to identify *C. parvum* and *C. hominis* with an LOD of 10 pg./μL of gDNA. Rahman et al. (2017) developed a LAMP method to detect *C. sinensis* with an LOD of 0.1 pg./μL gDNA or one egg/100 mg of feces. Moreover, Nkouawa et al. (2009) achieved an LOD of a single copy for detecting *T. solium* plasmid DNA. With the gradual maturation of the commercial application of microfluidic chip technology, LAMP combined with microfluidic chips has also been used for the detection of veterinary pathogens. Mao et al. (2018) demonstrated a multiplex microfluidic LAMP (mμLAMP) array for detecting *Anopheles* and *Plasmodium* spp. with an LOD of 1 pg. gDNA per reaction. Choi et al. (2016) developed a molecular diagnostic system (AnyMDx) based on LAMP technology to detect *Plasmodium falciparum* in the blood and achieved an LOD of 0.6 parasite per μL. Zhou et al. (2020) developed a microfluidic chip coupled with RT-LAMP for detecting porcine epidemic diarrhea virus (PEDV), porcine deltacoronavirus (PDCoV), and swine acute diarrhea syndrome-coronavirus (SADS-CoV), with LODs of 10^1^, 10^2^, and 10^2^ copies/μL of *in vitro*-transcribed RNA standards, respectively. In our previous research, we established an on-chip LAMP method for the detection of ten aquatic pathogenic bacteria, achieving an LOD of 0.40–6.42 pg. of gDNA per reaction (Zhou et al., 2014). In this study, we developed an on-chip LAMP assay capable of identifying five parasitic pathogens, highlighting the robust negative linear relationship between Tp and recombinant plasmid DNA quantities (Figure 4). Remarkably, this method demonstrated an LOD of 10^−2^ to 10^−3^ pg./μL of recombinant plasmid DNA (Figure 3). As mentioned earlier, the primer set Cpar-gp60 demonstrated a lower LOD compared to the primer set Cpar-SAM1 when using *C. parvum* gDNA as the template (Bakheit et al., 2008). However, in this study using on-chip LAMP assays, both the Cpar-gp60 and Cpar-SAM1 primer sets showed similar LODs when using recombinant plasmid DNA as the template (Figure 3). The observed difference in the LODs may be attributed to inherent variations in the quantities of both target genes within *Cryptosporidium* spp. Furthermore, the absence of loop primers in the Tsol-clp primer set (Nkouawa et al., 2009) resulted in higher Tp values when various concentrations of recombinant plasmid DNA were used as templates. For instance, at LOD concentrations, the Tp value approached 35 min (Figure 5).

Given the substantial decrease in individual reaction volumes on microfluidic chips compared to LAMP reactions in tubes, reproducibility is a key indicator of the reliability of microfluidic chip results. Zhou et al. (2020) used microfluidic chips (5 μL/reaction) in combination with RT-LAMP to detect PEDV, PDCoV, and SADS-CoV, achieving CV values ranging from 2.52–3.43%, 1.78–2.29%, and 2.59–3.75% when different concentrations of DNA were used as templates. Xu et al. (2022) developed an on-chip LAMP technique (10 μL/reaction) to detect multiple pathogens, including *Streptococcus agalactiae*, *Enterococcus faecalis*, *Gardnerella vaginalis*, *Candida albicans*, and *Chlamydia trachomatis*, in the lower genital tract during pregnancy, with CV values of 7.96, 6.63, 9.69, 7.56, and 9.87%, respectively, when a three-fold LOD concentration of DNA was used as a template. Hu et al. (2023) used an on-chip LAMP assay (5 μL/reaction) to identify three shrimp pathogens, *E. hepatopenaei*, white spot syndrome virus, and decapod iridescent virus 1, and achieved CV values ranging from 2.04–3.99%, 2.25–6.73%, and 3.87–9.59%, respectively, when different concentrations of recombinant plasmid DNA was used as the template. In this study, we examined the reproducibility of on-chip LAMP reactions using recombinant plasmid DNA as the template at concentrations close to the median. The results were remarkably consistent for each target gene, both within and across chips, as the Tp values of all reactions exhibited no significant differences (Figure 6). This finding highlights the high repeatability of the on-chip LAMP assays, particularly at higher template concentrations. When using DNA templates at LOD concentrations, all detection targets in the on-chip LAMP assays obtained positive results, albeit with some fluctuations. For instance, when pMD-Chom-SAM1 was used at the LOD concentration, the CV value for Tp exceeded 10%, indicating variability among replicates at lower DNA concentrations (Figure 5). Also using primer set Cpar-SAM1, the Tp value obtained using the DNA of recombinant plasmid pMD-Cpar-SAM1 at the LOD concentration was slightly lower than that obtained using the DNA of pMD-Chom-SAM1 at the LOD concentration (Figure 5). Moreover, the CV of the Tp values derived from pMD-Cpar-SAM1 was also lower than that derived from pMD-Cpar-SAM1 (Figure 5). These findings suggest that the Cpar-SAM1 primer set demonstrated better reproducibility for the DNA of pMD-Cpar-SAM1. This discrepancy is primarily attributable to the genetic polymorphism of SAM1 among *Cryptosporidium* spp. (Bakheit et al., 2008). Additionally, in the parallel detection of multiple DNA using templates with concentrations 10 times higher than the LOD, all targets achieved the expected positive results; however, the CV values for Tp for certain target genes fluctuated based on the mixed DNA (Figure 7). These findings suggest that the on-chip LAMP assays are highly reproducible, albeit with slight variability in the results.

Clinical sensitivity and specificity are important criteria in assessing the practicality of detection technologies. In detecting *C. sinensis*, the LAMP method showed a sensitivity of 97.1% and specificity of 100% in clinical tests (Rahman et al., 2017). Zhao et al. (2010) developed a LAMP method to detect *Escherichia coli* O_157_ in food samples, achieving a clinical sensitivity of over 96.3% and specificity of 100%. The LAMP-based detection system developed by Mao et al. (2018) also exhibited a high sensitivity (>95%) and specificity (100%). The RT-LAMP-based microfluidic chip developed by Zhou et al. (2020) showed sensitivity values of 92.24, 92.19, and 91.23% for PEDV, PDCoV, and SADS-CoV, respectively, and a clinical specificity of 100%. In our previous study, we reported an on-chip LAMP assay for the detection of ten pathogenic bacteria, achieving a clinical sensitivity of 96.2% and clinical specificity of 93.8% (Zhou et al., 2014). In this study, the clinical sensitivity and specificity of the on-chip LAMP method were 98.08 and 97.59%, respectively (Table 2), which were close to those of the PCR method (94.23 and 98.80%, respectively). In public places such as farmers’ markets, the presence of a variety of raw foods, including freshwater products, meats, and vegetables, increases the risk of cross-contamination by foodborne parasites like *T. gondii*, *Cryptosporidium* spp., *C. sinensis*, and *T. solium* (Dorny et al., 2009; Carrique-Mas and Bryant, 2013; Ng-Nguyen et al., 2018; Li et al., 2020; da Costa Dantas et al., 2023). The high clinical sensitivity and specificity of the on-chip LAMP method make it an effective tool for screening and monitoring foodborne parasites in raw foods from different sources, especially in public settings.

The costs of nucleic acid-based molecular diagnostic systems primarily constitute the expenses of reagents and devices. Compared to other molecular biology assays, the LAMP method incurs lower costs (Zhou et al., 2014; World Health Organization, 2016). Although the production of LAMP reagents has matured, their price has not significantly decreased compared to that described in our previous study (Zhou et al., 2014). Among LAMP-based detection systems, variations in reagent costs mainly depend on the reagent dosage during isothermal amplification. To perform a LAMP assay in a polypropylene tube, the estimated cost of the reagents would be $ 416 for 32 LAMP reactions using a standard reaction volume of 25 μL (Table 3). In this study, the calculated reagent cost for 32 LAMP reactions on a whole chip was no more than $ 104 (Table 3), owing to the reduced volume of 5 μL per reaction. Therefore, the estimated cost of the reagents for detecting one sample was approximately $ 26, while the cost for detecting one targeted nucleic acid is approximately $ 3.25. Isothermal amplification-based systems have made significant progress in many aspects, such as portability and intelligence. For example, compared to CapitalBio RTisochip™-A, RTisochip™-C has added an LCD display, no longer requiring an external computer, and has been equipped with a mobile power supply, making it highly portable (Table 3; Zhou et al., 2014; Lin et al., 2022). Other detection systems, such as the ESEQuant Tube Scanner (Qiagen Lake Constance GmbH, Stockach, Germany) and Genie III (OptiGene, Horsham, United Kingdom), feature portability (Table 3; Njiru, 2012; https://www.optigene.co.uk/instruments/instrument-genie-iii). The iGeneTec™ SC-MA2000 used in this study features portability and the ability to function without an external computer. These optimizations and functionality upgrades come at an unchanged price (Table 3). To promote the adoption of isothermal amplification-based methods, several companies have introduced comprehensive marketing strategies that bundle chips and compatible instruments together. For example, the CapitalBio RTisochip™-A system offers free instruments when purchasing 5,000 chips (Table 3; Zhou et al., 2014). In this study utilizing on-chip LAMP assays, the iGeneTec™ SC-MA2000 device was provided free of charge, along with lifelong technical service and maintenance support, if a minimum of 1,000 chips were purchased annually, with no additional fees (Table 3). Such initiatives significantly reduce the overall cost of implementing this method, showcasing promising prospects for widespread adoption. Moreover, the chip’s design can be customized to include flexible arrays of reaction wells in configurations like 2 × 16, 4 × 8, or 8 × 4, enhancing its practicality. Nucleic acid extraction-integrated detection systems, such as the EasyNAT® System, have been commercialized, streamlining the testing process for simple sample types such as blood and urine, despite being expensive (Table 3; Cassa-Loaiza et al., 2022). AnyMDx, another LAMP-based system integrated with nucleic acid extraction, was predicted to cost only $ 176 for the prototype device and $ 1.14 per reaction for the reagent (Choi et al., 2016); however, it remains commercially unavailable. These accomplishments underscore significant room for improvement in the development of isothermal amplification-based microfluidic chip technology.

**Table 3:** Comparison of several LAMP or microfluidic LAMP systems.

Device	Device cost ($)	Commercialization status of the device	cost of 32 reactions ($)	Portability	Integrated with nucleic acid extraction	Sample operation	References
iGeneTec™ SC-MA2000	11, 000	Yes	104	Yes	No	One-off loading by a pipettor per sector on a chip	In this study
CapitalBio RTisochip™-C	15, 000	Yes	33	Yes	No	One-off loading by a pipettor and short centrifugation	Lin et al. (2022)
Deaou-308C	28, 000	Yes	416	No	No	Loading in 200 μL polypropylene tube one by one	Ma et al. (2020)
ESEQuant Tube Scanner	23, 200	Yes	416	Yes	No	Loading in 200 μL polypropylene tube one by one	Njiru (2012)
EasyNAT® System	43, 000	Yes	>416	No	Yes	One-off loading in a customized tube one by one	Cassa-Loaiza et al. (2022)
AnyMDx system	176	No	36.5	Yes	Yes	One-off loading by a pipettor per unit on a chip	Choi et al. (2016)
Genie III	20,045	Yes	416	Yes	No	loading in 200 μL polypropylene tube one by one	https://www.optigene.co.uk/instruments/instrument-genie-iii

## 5. Conclusion

In this study, we developed an on-chip LAMP method capable of identifying five zoonotic parasites: *T. gondii*, *C. parvum*, *C. hominis*, *C. sinensis*, and *T. solium*. This LAMP assay is performed on a centrifugal microfluidic chip that limits the volume of each reaction to 5 μL, providing a highly automated format for examining up to four samples simultaneously. The on-chip LAMP reaction was performed at 65°C for 60 min, achieving excellent clinical sensitivity and specificity for field sample detection with minimal labor. Overall, this on-chip LAMP strategy provides a convenient and reliable platform for the on-site detection and routine monitoring of foodborne parasites. It is essential to include the detection of other foodborne parasites in this on-chip LAMP platform in future studies. This will effectively aid disease prevention and control agencies in monitoring foodborne parasites in complex public settings or environments to prevent the spread of foodborne parasitic diseases.

## Data availability statement

The original contributions presented in the study are included in the article/Supplementary material, further inquiries can be directed to the corresponding authors.

## Ethics statement

The animal study was approved by Animal Ethics and Welfare Committee (AEWC) of Ningbo University. The study was conducted in accordance with the local legislation and institutional requirements.

## Author contributions

Y-XC and Y-RL: investigation, visualization, and writing original draft. L-JD, Z-JX, F-JC, H-XC, and G-ZX: investigation and visualization. Q-JZ: conceptualization, methodology, visualization, writing-reviewing and editing original draft, supervision, funding acquisition. A-FD: resources, reviewing & editing original draft. JC: reviewing & editing original draft, funding acquisition. All authors contributed to the article and approved the submitted version.

## Funding

The author(s) declare financial support was received for the research, authorship, and/or publication of this article. The work was supported by the Key Research and Development Project of Zhejiang Province (2021C02062, 2021C02059), the Program of Science and Technology Department of Zhejiang Province (LGN22C010001), National Natural Science Foundation of China (42276110), Natural Science Foundation of Ningbo City, China (2021 J061), the Program of Science and Technology Department of Ningbo City (2022S210), State Key Laboratory for Managing Biotic and Chemical Threats to the Quality and Safety of Agro-products (2021DG700024-ZZ2102, 2021DG700024-KF202219), One health Interdisciplinary Research Project, Ningbo University (HZ202201), Zhejiang Key Laboratory of Exploitation and Preservation of Coastal Bio-resource (J2022001), the Program of Zhejiang Agriculture and Rural Affairs (2023SNJF062).

## Conflict of interest

The authors declare that the research was conducted in the absence of any commercial or financial relationships that could be construed as a potential conflict of interest.

## Publisher’s note

All claims expressed in this article are solely those of the authors and do not necessarily represent those of their affiliated organizations, or those of the publisher, the editors and the reviewers. Any product that may be evaluated in this article, or claim that may be made by its manufacturer, is not guaranteed or endorsed by the publisher.

## Supplementary material

SUPPLEMENTARY FIGURE 1 The amplification curves for reproducibility analysis of on-chip LAMP reactions. Eight reaction wells from one sector of a chip were pre-immobilized with a unique primer set and pipetted with a same amount of DNA from one recombinant plasmid. DNA (1 pg/μL) from each recombinant plasmid was used as the template. Sterile, DNA-free ddH_2_O was employed as a negative control. **(A)** Primer set: Tgon-repeatregion, Template: pMD-Tgon-repeatregion; **(B)** Primer set: Cpar-gp60, Template: pMD-Cpar-gp60; **(C)** Primer set: Cpar-SAM1, Template: pMD-Cpar-SAM1; **(D)** Primer set: Cpar-SAM1, Template: pMD-Chom-SAM1; **(E)** Primer set: Csin-cathepsin, Template: pMD-Csin-cathepsin; **(F)** Primer set: Tsol-clp, Template: pMD-Tsol-clp.
