# Supplementary figures and images for: Parallel detection of multiple zoonotic parasites using a real-time fluorogenic loop-mediated isothermal amplification-based quadruple-sample microfluidic chip
Source: Front Microbiol. 2023 Sep 26;14:1238376. doi: 10.3389/fmicb.2023.1238376 (PMC10562543; doi:10.3389/fmicb.2023.1238376)

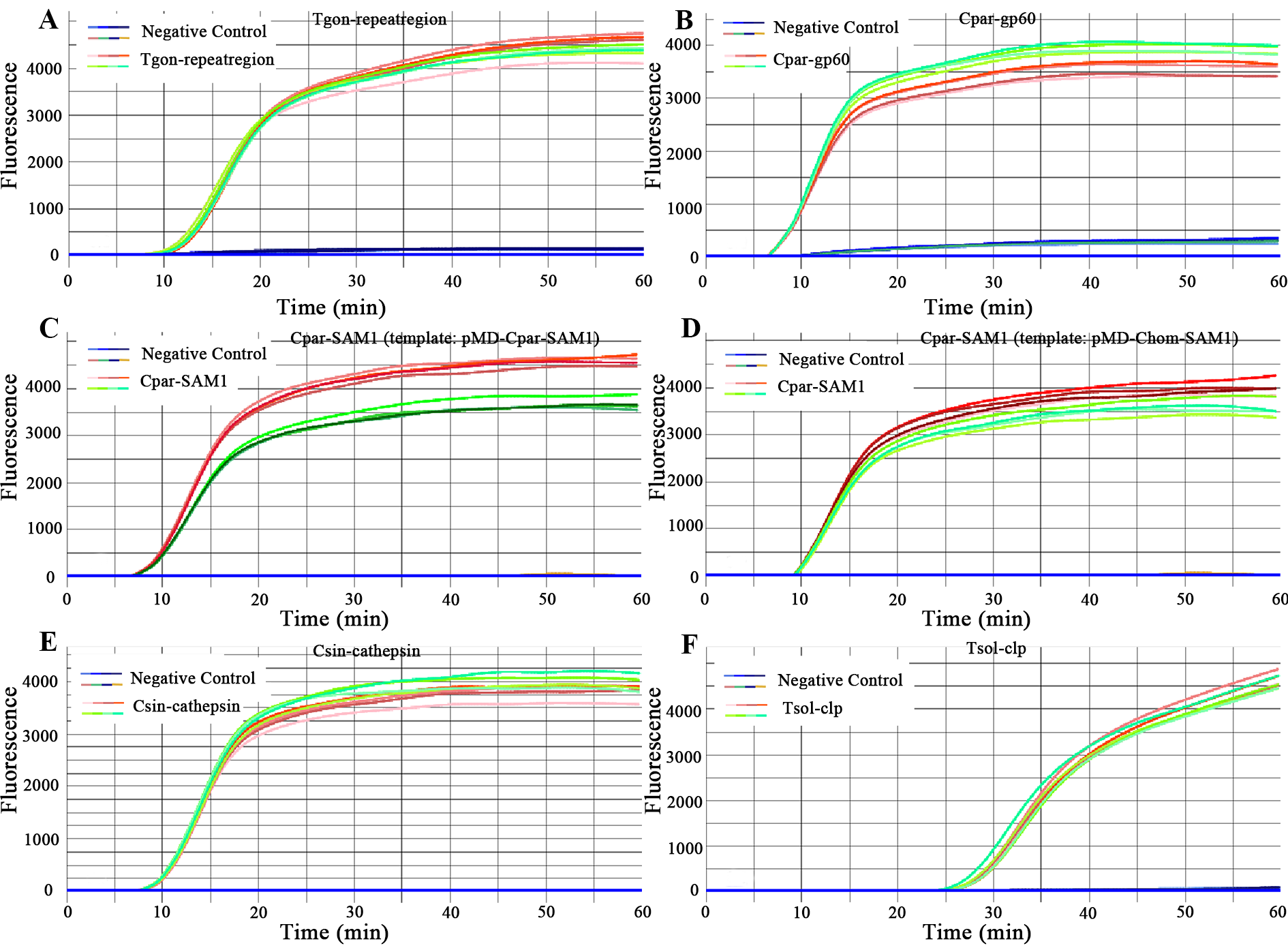

Supplement: SUPPLEMENTARY FIGURE 1 — The amplification curves for reproducibility analysis of on-chip LAMP reactions. Eight reaction wells from one sector of a chip were pre-immobilized with a unique primer set and pipetted with a same amount of DNA from one recombinant plasmid. DNA (1 pg/μL) from each recombinant plasmid was used as the template. Sterile, DNA-free ddH2O was employed as a negative control. (A) Primer set: Tgon-repeatregion, Template: pMD-Tgon-repeatregion; (B) Primer set: Cpar-gp60, Template: pMD-Cpar-gp60; (C) Primer set: Cpar-SAM1, Template: pMD-Cpar-SAM1; (D) Primer set: Cpar-SAM1, Template: pMD-Chom-SAM1; (E) Primer set: Csin-cathepsin, Template: pMD-Csin-cathepsin; (F) Primer set: Tsol-clp, Template: pMD-Tsol-clp. [file Image_1.tif]
