# Supplementary material for: Parallel detection of multiple zoonotic parasites using a real-time fluorogenic loop-mediated isothermal amplification-based quadruple-sample microfluidic chip
Source: Front Microbiol. 2023 Sep 26;14:1238376. doi: 10.3389/fmicb.2023.1238376 (PMC10562543; doi:10.3389/fmicb.2023.1238376)
Supplement: Supplementary file 2 [file Table_1.docx]

Supplementary Table 1 Zoonotic parasitic and bacterial isolates used in this study

| Parasitic/Bacterial isolates | Isolate No. | Source | Culture conditions |
| --- | --- | --- | --- |
| *Toxoplasma gondii* | type II strain ME49 | Provided by Prof. Du Aifang (College of Animal Sciences, Zhejiang University, China) | Maintained on monolayers of African green monkey kidney (Vero) cells at 37 °C and 5% CO2 in Dulbecco’s Modified Eagle’s Medium (DMEM, HyClone) supplemented with 10% fetal calf serum (FCS, HyClone), 2 mM L-glutamine, and 100 units of penicillin/100 mg of streptomycin (Sun et al., 2017) |
| *Cryptosporidium parvum* | - |  | The oocysts were preserved in 2.5% potassium dichromate at 4 °C, following the method described by Yang et al. (2016) |
| *Cryptosporidium hominis* | - |  |  |
| *Cryptosporidium suis* | - |  |  |
| *Cryptosporidium baileyi* | ZJ stain |  |  |
| *Toxocara canis* | - |  | - |
| *Clonorchis sinensis* | - |  | - |
| *Taenia solium* | - |  | - |
| *Trichinella spiralis* | - |  | - |
| *Ichthyophthirius* sp. | - | Provided by Prof. Wang Qing [The Pearl River Fisheries Research Institute (PRFRI), Chinese Academy of Fishery Sciences (CAFS)] | - |
| *Anisakis simplex* | - | Isolated and kept in our lab | Stored in absolute alcohol at -20 ℃ (Qiao et al., 2019) |
| *Enterocytozoon hepatopenaei* | - | Provided by Dr. Yan Maocang (Zhejiang Mariculture Research Institute, China) | Infected animal specimens were confirmed using PCR combined with histological staining, as described in a previous study (Tang et al., 2015) |
| *Babesia bovis* | - | Provided by Prof. Du Aifang | - |
| *Schistosoma japonicum* | - |  | - |
| *Glugea plecoglossi* | - | Isolated and kept in our lab | Isolated from the diseased *Plecoglossus altivelis* by crushing the xenomas and releasing the spores in normal saline (Zhou et al., 2018) |
| *Streptococcus iniae* | ATCC 29178 | Purchased from American Type Culture Collection (ATCC) | Grown on trypticase soy agar or in broth (TSA or TSB) at 30 °C for 24 h or 12 h |
| *Aeromonas hydrophila* | ATCC 7966 | Purchased from China General Microbiological Culture Collection Center (CGMCC) | Cultured in Luria-Bertani broth (LB) overnight at 37 °C (Zhou et al., 2021) |
| *Vibrio Parahemolyticus* | ATCC 33845 | Provided by Prof. Zhang Yanjun (Zhejiang Provincial Center for Disease Control and Prevention, China) | Cultured overnight at 30 °C using tryptic soy broth (TSB) or thiosulfate-citrate-bile salt-sucrose (TCBS) agar |
| *Listeria monocytogenes* | ATCC 13444 | Purchased from ATCC | Cultured on brain-heart infusion (BHI) broth overnight at 37 ℃ |

**References**

Qiao, Y., Zhou, Q.-J., Li, X.-J., Miao, L., and Chen, J. (2019). A loop-mediated isothermal amplification technique combined with a lateral flow dipstick for the detection of *Annisakis simplex* sensu stricto/*anisakis pegreffii* in commercial fish. *Oceanologia Et Limnologia Sinica (in Chinese)* 5: 324-335. doi: 10.11693/hyhz20180800207.

Sun, H., Zhuo, X., Zhao, X., Yang, Y., Chen, X., Yao, C., et al. (2017). The heat shock protein 90 of *Toxoplasma gondii* is essential for invasion of host cells and tachyzoite growth. *Parasite* 24: 22. doi: 10.1051/parasite/2017023.

Tang, K.F.J., Pantoja, C.R., Redman, R.M., Han, J.E., Tran, L.H., and Lightner, D.V., (2015). Development of in situ hybridization and PCR assays for the detection of *Enterocytozoon hepatopenaei* (EHP), a microsporidian parasite infecting penaeid shrimp. *J. Invertebr. Pathol.* 130: 37-41. doi: 10.1016/j.jip.2015.06.009.

Yang, Y., Xue, X., Yang, Y., Chen, X., and Du, A. (2016). Efficacy of a potential DNA vaccine encoding *Cryptosporidium baileyi* rhomboid protein against homologous challenge in chickens. *Vet. Parasitol.* 225: 5-11. doi: 10.1016/j.vetpar.2016.05.024.

Zhou, Q.J., Chai, F.C., and Chen, J. (2018). First record of *Glugea plecoglossi* (Takahashi & Egusa, 1977), a microsporidian parasite of ayu (*Plecoglossus altivelis altivelis* Temminck & Schlegel, 1846) in Mainland China. *J. Fish. Dis.* 41: 165-169. doi: 10.1111/jfd.12674.

Zhou, Q.-J., Lu, J.-F., Su, X.-R., Jin, J.-L., Li, S.-Y., Zhou, Y., et al. (2021). Simultaneous detection of multiple bacterial and viral aquatic pathogens using a fluorogenic loop‐mediated isothermal amplification‐based dual‐sample microfluidic chip. *J. Fish. Dis.* 44: 401-413. doi: 10.1111/jfd.13325.
