# Supplementary material for: Parallel detection of multiple zoonotic parasites using a real-time fluorogenic loop-mediated isothermal amplification-based quadruple-sample microfluidic chip
Source: Front Microbiol. 2023 Sep 26;14:1238376. doi: 10.3389/fmicb.2023.1238376 (PMC10562543; doi:10.3389/fmicb.2023.1238376)
Supplement: Supplementary file 3 [file Table_2.docx]

Supplementary Table 2 Reproducibility analysis of LAMP reactions within and between chips

| Target gene | Statistical significance analysis of Tp values within a chip | | | | Statistical significance analysis of Tp values between 3 chips | | |
| --- | --- | --- | --- | --- | --- | --- | --- |
|  | Chip set No. | Source of variation | df | P value | Source of variation | df | P value |
| Tgon-repeatregion | 1 | between units | 1 | 0.900 | between chips | 2 | 0.425 |
|  |  | within units | 6 |  |  |  |  |
|  |  | total variation | 7 |  |  |  |  |
|  | 2 | between units | 1 | 0.502 | within chips | 21 |  |
|  |  | within units | 6 |  |  |  |  |
|  |  | total variation | 7 |  |  |  |  |
|  | 3 | between units | 1 | 0.203 | total variation | 23 |  |
|  |  | within units | 6 |  |  |  |  |
|  |  | total variation | 7 |  |  |  |  |
| Cpar-gp60 | 1 | between units | 1 | 1.000 | between chips | 2 | 0.060 |
|  |  | within units | 6 |  |  |  |  |
|  |  | total variation | 7 |  |  |  |  |
|  | 2 | between units | 1 | 0.490 | within chips | 21 |  |
|  |  | within units | 6 |  |  |  |  |
|  |  | total variation | 7 |  |  |  |  |
|  | 3 | between units | 1 | 0.399 | total variation | 23 |  |
|  |  | within units | 6 |  |  |  |  |
|  |  | total variation | 7 |  |  |  |  |
| Cpar-SAM1 (template: pMD-Cpar-SAM1) | 1 | between units | 1 | 1.000 | between chips | 2 | 0.229 |
|  |  | within units | 6 |  |  |  |  |
|  |  | total variation | 7 |  |  |  |  |
|  | 2 | between units | 1 | 0.522 | within chips | 21 |  |
|  |  | within units | 6 |  |  |  |  |
|  |  | total variation | 7 |  |  |  |  |
|  | 3 | between units | 1 | 0.320 | total variation | 23 |  |
|  |  | within units | 6 |  |  |  |  |
|  |  | total variation | 7 |  |  |  |  |
| Cpar-SAM1 (template: pMD-Chom-SAM1) | 1 | between units | 1 | 1.000 | between chips | 2 | 0.122 |
|  |  | within units | 6 |  |  |  |  |
|  |  | total variation | 7 |  |  |  |  |
|  | 2 | between units | 1 | 1.000 | within chips | 21 |  |
|  |  | within units | 6 |  |  |  |  |
|  |  | total variation | 7 |  |  |  |  |
|  | 3 | between units | 1 | 0.730 | total variation | 23 |  |
|  |  | within units | 6 |  |  |  |  |
|  |  | total variation | 7 |  |  |  |  |
| Csin-cathepsin | 1 | between units | 1 | 0.107 | between chips | 2 | 0.107 |
|  |  | within units | 6 |  |  |  |  |
|  |  | total variation | 7 |  |  |  |  |
|  | 2 | between units | 1 | 0.067 | within chips | 21 |  |
|  |  | within units | 6 |  |  |  |  |
|  |  | total variation | 7 |  |  |  |  |
|  | 3 | between units | 1 | 0.215 | total variation | 23 |  |
|  |  | within units | 6 |  |  |  |  |
|  |  | total variation | 7 |  |  |  |  |
| Tsol-clp | 1 | between units | 1 | 0.216 | between chips | 2 | 0.511 |
|  |  | within units | 6 |  |  |  |  |
|  |  | total variation | 7 |  |  |  |  |
|  | 2 | between units | 1 | 0.805 | within chips | 21 |  |
|  |  | Within units | 6 |  |  |  |  |
|  |  | total variation | 7 |  |  |  |  |
|  | 3 | between units | 1 | 0.083 | total variation | 23 |  |
|  |  | within units | 6 |  |  |  |  |
|  |  | total variation | 7 |  |  |  |  |

Note: All data are described as means±standard error of the mean (SEM). Data were analyzed by one-way analysis of variance (ANOVA) using SPSS v22.0 (SPSS Inc, Chicago, IL, USA). A P-value of 0.05 was considered statistically significant.
